# Supplementary material for: Communicating a Plan for Involuntary Psychiatric Admission: A Standardized Patient Workshop Intervention for General Psychiatry Residents
Source: MedEdPORTAL. 2023 Oct 17;19:11355. doi: 10.15766/mep_2374-8265.11355 (PMC10579457; doi:10.15766/mep_2374-8265.11355)
Supplement: Supplementary file 1 — Needs Assessment Survey.docxPSTLC Protocol.docxWorkshop Scenario Door Prompts.docxSP Case Development Tool.docxPreworkshop Survey.docxProtocol Feedback Checklist.docxPostworkshop Survey.docx [file mep_2374-8265.11355-s001.zip › A. Needs Assessment Survey.docx]

**Appendix A. NEEDS ASSESSMENT SURVEY**

1. Have you ever had formal teaching on how to tell a patient that they are involuntarily committed?

[ ] Yes

[ ] No

If yes, please describe what kind of teaching you have had.

_____________________________________________________________________________

1. Have you ever purposely avoided telling a patient they are involuntarily committed because you were afraid of how they would react?

[ ] yes

[ ] no

[ ] I don’t remember

1. How comfortable are you with your ability to tell a patient that they are being involuntarily committed?

[ ] Not at all comfortable

[ ] A little bit comfortable

[ ] Somewhat comfortable

[ ] Quite comfortable

[ ] Very comfortable

1. What are you not comfortable about? Rate the below statements from 0 to 100, where 0 = not true at all, 50 = somewhat true, and 100 = absolutely true.

___ I have difficulty determining the appropriate words to use to break the news of involuntary commitment to patients.

___ ] I have difficulty determining the amount of information to share with the patient about the involuntary commitment process

___ My lack of familiarity with the involuntary commitment process limits my ability to talk about it with the patient.

___ I fear that arguments from the patient about involuntary commitment will make me uncertain of my decision.

___ I fear that discussions about involuntary commitment will lead to patient violence or agitation.

___ I dislike giving “disappointing news.”

1. What else are you not comfortable about? ____________________________________________________________________________________________________________________________________________________________

1. I believe training in involuntarily commitment should be a part of my residency training curriculum.

[ ] Strongly agree

[ ] Agree

[ ] Neutral

[ ] Disagree

[ ] Strongly disagree

What questions or comments do you have about telling a patient they are involuntarily committed?

__________________________________________________________________________________________________________________________________________________________________________________________________________________________________________

**DEMOGRAPHICS**

1. What sex/gender do you identify as?

[ ] Male

[ ] Female

[ ] Other ____________________________________________

1. Level of training

[ ] PGY-1

[ ] PGY-2

[ ] PGY-3

[ ] PGY-4

1. What future practice setting do you envision yourself in? (choose all that apply)

[ ] Outpatient

[ ] Inpatient

[ ] Emergency

[ ] Consult

[ ] Community

[ ] >50% nonclinical (e.g. administrative, research, education)

[ ] Undecided

[ ] Other _____________________________________________

1. How important do you think learning to tell a patient they are being involuntarily committed is in your future practice?

[ ] Not at all important

[ ] Somewhat important

[ ] Moderately important

[ ] Very important

[ ] Extremely important
