# Supplementary material for: Communicating a Plan for Involuntary Psychiatric Admission: A Standardized Patient Workshop Intervention for General Psychiatry Residents
Source: MedEdPORTAL. 2023 Oct 17;19:11355. doi: 10.15766/mep_2374-8265.11355 (PMC10579457; doi:10.15766/mep_2374-8265.11355)
Supplement: Supplementary file 1 — Needs Assessment Survey.docxPSTLC Protocol.docxWorkshop Scenario Door Prompts.docxSP Case Development Tool.docxPreworkshop Survey.docxProtocol Feedback Checklist.docxPostworkshop Survey.docx [file mep_2374-8265.11355-s001.zip › B. PSTLC Protocol.docx]

# **Appendix B. P.S. TLC PROTOCOL:**

# **Delivering Involuntary Commitment News**

1. **Initial intake**

- Familiarize yourself with the state commitment laws.
- Start interviews open-ended.
- LISTEN. 80% of the conversation is listening. Get their understanding of why they are here.
- Get collateral if warranted.
- After you’ve made your decision to commit the patient, follow steps below.

1. **PREP & Safety**

Safety

- Ensure prns are ordered if concern for significant agitation/aggression.
- Inform ancillary staff that you are going to have this conversation (includes nursing staff, assessment specialist, consider police/security) so they are aware and can draw up medications/look for signs of help if needed.

Timing

- Best to time the conversation to when they are ready to be moved to next episode of care, or as late as the interviewing provider can do this.
  - Another viewpoint is to tell the patient as early as possible to avoid running into issues of agitation during transport.
- Ensure all admission orders are in, legal documents are completed.
- Always have this conversation before admission. It is better for the floor staff and the primary team. Also, the emergency setting is best equipped to deal with situations like this.

Location

- Always ensure a place for private conversation is available before going to speak with the patient.
- Ask the patient where they want to talk.
- Consider keeping the door open and having the conversation at the doorway if concerned for safety.

Provider

- The main provider who interviewed and assessed the patient should be the one to speak to the patient.
  - Consults: consider having the primary team in the room to avoid splitting.
- Make sure you are clear about the reasons that you are committing the patient. Any ambivalence or uncertainty will come out in your conversation!
- Brainstorm any contingencies that will cause you to change your mind. I.e., ask yourself, what things might the patient say that will make me think commitment is not necessary?
  - If the patient insists on leaving, consider having the patient come up with a safety plan. If it is not adequate, then involuntary commitment may be necessary.

1. **SUMMARIZE concerns and state rationale**

- Principle: First, make sure you clearly express you are coming from a place of concern and desire to help. Second, convincingly and transparently describe the rationale.
- Summarizing helps the patient know you are on the same page in terms of the presenting facts of the case.
- Be explicit about the grounds on which you are committing the patient. Lack of knowledge about what is happening and why causes patents to feel they are losing control and spark agitation.
- Avoid jargon terms and use terminology to meet the patient where they are in terms of understanding.

1. **Be TRANSPARENT and clearly state involuntary commitment decision**

- Be transparent about the commitment decision. Do not be ambivalent.
- Convey the message that this is NOT punishment, this is about safety**.**

1. **LISTEN, then Empathize…**

- Do not overlook or ignore the patient’s concerns, as that will be a reason for agitation. Therapeutic alliance is still important, especially in this setting.
- Build the standpoint of wanting to work together with the patient.
- Make sure you understand their side of the story. Remind them of their motivations for treatment if applicable.
- Patients are often frightened by the feeling of powerlessness. Let them know what the influences are that will control their length of stay (i.e., ensuring safety and/or stability, outpatient plan is in place, etc.). However, never make promises of how long they will be in the hospital.

**4b. …and Re-emphasize your decision**

- Make sure they understand your decision-making process. Repeat the reason they came in the first place, and your rationale.
- Can confront with conflicting collateral information, however avoid shifting blame onto them as maintaining a positive relationship with family members/close supports is just as important (if not more) to the patient’s care.
- Answer any questions they have. Uncertainty is a cause of fear and agitation.
- Empathy and transparency should be evident throughout the conversation.
- Keep conversation short.

1. **Post-conversation COMMUNICATION**

- Communicate to the receiving nurse/team any safety concerns.
- Let the patient know you’re available if they want to talk or have questions.
- Check up on them occasionally if they are delays in transport – the focus of the latter two points is to emphasize coming from a position of concern/care and safety.

# **EXAMPLE QUOTES FOR EACH STEP**

| **Step** | **Example quotes** |
| --- | --- |
| 1. Initial intake | “What’s your understanding of being in the hospital?” |
| 1. Prep & Safety |  |
| 1. Summarize concerns and state rationale | “You came in to the hospital looking very distressed and wanting to run into traffic. This is something we take very seriously. I think this is the help you need.”  “I’m really worried about you.”  “I don’t want to risk waiting until you act on your suicidal thoughts before getting you treatment.”  “I’m really concerned that you’re telling me you can’t keep yourself safe, and I think this is the best way I have to make sure that you can stay safe and start to feel better.”  “I need to make sure that you can be safe when you leave, and I’m worried because what you’ve said/done showed me that you’re not right now” |
| 1. Be transparent and clearly state involuntary commitment decision | “Based on my assessment, I think you need to be in the hospital whether or not you believe you need to be, and this is why.” If they refuse – “in these specific situations, we actually have the ability to temporarily suspend your right to do that while we are ensuring you are getting proper treatment/for safety reasons.”  “I understand you want to leave the hospital, but at this moment in time, that’s not possible.”  “If we are this concerned, we have the ability to admit you to the hospital even without your consent, in order to ensure your safety.”  “I have an obligation to take care of you, and I’m not going to let you down by putting you in a risky situation when I know I can help.”  “This is not a punishment; this is about your safety.” |
| 4a. Listen, then Empathize… | “I hear you; at the same time we also have this different side of the story from [collateral]. In order to help us make this decision, we need to know you better and observe you for a period of time.”  “Coming to the hospital is not fun for anyone but we will do everything we can to get you out as quickly as possible, as soon as we can ensure safety.”  “I know it’s not pleasant, but we are trying to understand you so that we can help in every way we can.”  “This isn’t something where you’re going to be locked away for months and months. Our goal is to [state specific goal], and once we’re there, there won’t be a need for you staying in the hospital.”  “You clearly have a lot of people that care about you, and we shouldn’t let them or you down by missing an opportunity to help” |
| 4b. …and Re-emphasize your decision | “Do you remember when we were talking about your suicidal thoughts [other reasons for commitment]? These are things we take very seriously, and under these circumstances, I will need to insist on giving you the appropriate care you need and that we are able to provide.”  “At this point based on what you’ve told me already, I have to insist on this decision from a safety perspective, and I wouldn’t be doing so if I didn’t think there would be a lot of benefit from doing so.”  If the conversation appears to be going in circles: “I understand this is not what you want, but I don’t think we’re going to be able to find common ground here and this is what will need to happen.” |
| 5. Post-conversation communication |  |
