# Supplementary material for: Communicating a Plan for Involuntary Psychiatric Admission: A Standardized Patient Workshop Intervention for General Psychiatry Residents
Source: MedEdPORTAL. 2023 Oct 17;19:11355. doi: 10.15766/mep_2374-8265.11355 (PMC10579457; doi:10.15766/mep_2374-8265.11355)
Supplement: Supplementary file 1 — Needs Assessment Survey.docxPSTLC Protocol.docxWorkshop Scenario Door Prompts.docxSP Case Development Tool.docxPreworkshop Survey.docxProtocol Feedback Checklist.docxPostworkshop Survey.docx [file mep_2374-8265.11355-s001.zip › C. Workshop Scenario Door Prompts.docx]

Appendix C. Workshop Scenario Door Prompts

**EXAMINEE INSTRUCTIONS**

**Case Name:** Case #1

1. **Opening Scenario**

- Patient name: Jenny Donlon
- Sex: Female
- Presents to: Local emergency psychiatry services
- Chief complaint: Mania

1. **Vital Signs**

- Temperature: 98°F
- Blood Pressure: 135/87 mmHg
- Pulse: 95 bpm
- Respirations: 13 breaths per min

1. **Case:**

Jenny Donlon is a 50-year-old female working as a middle-school teacher brought in to local emergency psychiatry services via police after her husband, Bobby Donlon, called community crisis sevices for bizarre behavior. When you had asked Jenny what brought her here, she stated that she was brought here by her family for "coming out as a lesbian, because they think that's related to mental health issues." She states how she found her "inner zen" and have plans to start a new spiritual and pure life with her new girlfriend. She was perseverative on how her family does not approve of her lifestyle, and how she is being held against her will. You had asked her multiple clarifying questions, but she is discharge-focused and repeats how she has no mental health issues and just wants to go home, particularly to “meet with my girlfriend, and probably future wife." She tells you that her outpatient psychiatrist cleared her of any psychiatric illnesses and told her to discontinue her medication, which she did several months ago.

When you asked if you can contact a family member or her girlfriend, she tells you that her girlfriend is Robyn Fenty (Rihanna), and her “true” family is Sean Carter (Jay-Z), and she calls these people her "advocates." You again ask about other family members, specifically her husband. She adamantly denies that Bobby is her husband, and that "I must leave him, because I'm lesbian. I'm planning to start a whole new life with Robyn."

You contacted Jenny’s husband, Bobby Donlon. He stated he was out of town for business over the weekend and Jenny was supposed to pick him up from the airport yesterday but never did. When he arrived home, she had her bags packed, saying she was waiting for a friend to pick her up to go to the airport to "fly somewhere," because her mother died. He confirmed that her mother did not die by calling her, however Jenny continued to demonstrate disorganized and bizarre behavior. He described her as "difficult to talk to" and "not making sense," as she stated that Social Security had owed her $12,000, and then attempted to go online to buy a laptop so that she can speak to "the higher people." He told you that during prior manic episodes, Jenny had a history of excessive traveling and spending; one time she had closed all of her bank accounts, then attempted suicide via carbon monoxide poisoning by locking herself in the car. He described the patient as "anti-medicine" with a history of frequent medication noncompliance.

**You are now preparing to go back to see the patient after you’ve completed your initial intake and called collateral. You would like to commit the patient for the following reasons:**

- **Pt has demonstrated risky behavior during episodes of mania (i.e., excessive spending, reckless traveling) that can lead to imminent risk of harm (e.g., financially, bodily, etc.) and further functional decline without treatment**
- **Husband has expressed that he is unable to keep his wife safe at home, thus there is no other safe alternative**

4. Examinee Tasks:

1. Talk through the preparatory steps you would take before you see the patient.
2. See the patient and inform them that they are being involuntarily committed.
3. Exit the interview when you think it is appropriate to do so

**EXAMINEE INSTRUCTIONS**

**Case Name:** Case #2

1. **Opening Scenario**

- Patient name: Melanie Walker
- Sex: Female
- Presents to: Local emergency psychiatry services
- Chief complaint: Paranoid delusions

1. **Vital Signs**

- Temperature: 98°F
- Blood Pressure: 140/83 mmHg
- Pulse: 76 bpm
- Respirations: 16 breaths per min

1. **Case:**

You are the resident in local psychiatry emergency services seeing Melanie Walker, a 60-year-old female with past medical history of hypertension, hyperlipidemia, and cerebrovascular accidents (recently 3 small strokes in the past year with mild residual left-sided weakness) brought in by her husband for progressively worsening delusions. Her husband stated that she had “completely lost touch with reality,” and she had not been able to care for herself anymore.

You first speak with Melanie privately. When you ask what brings her here, she had said, “They got him. They made him think I’ve got some mental problem. But I know the truth. And they don’t know that I know that. There’s nothing wrong with me mentally, so I came here to prove it to him.” Regarding her husband, she had stated, “The FBI are messing with me. They’ve sent this man. I’m not crazy. I’m normal. I won’t let them get to me. You got to protect yourself.” She denies any prior psychiatric history.

She consents for you to speak with her husband, Sammy Walker, privately. He states that Melanie had been previously working as a librarian but was let go in the past few months after she had several arguments with her coworkers; her manager had described her as “talking out of her head.” She has also had several verbal disagreements with Sammy at home which seem to be surrounding her concerns of a conspiracy against her by the CIA and FBI. Melanie states they are targeting her because she has “figured them out.” Sammy states she also has been keeping the window blinds closed and putting black tape over mirrors and ceiling fans. Most recently she had been making statements where she felt Sammy was now also being controlled by the FBI and being turned against her. In the past few days, she had been refusing to sleep in the bed at night and instead had moved to sleeping on the couch in the living room. He also states she had been eating less than usual, making comments that her food is poisoned and “they think I don’t know” and something about “invisible forces.” Sammy brought her to her neurologist’s office yesterday for these concerns, and the neurologist called back earlier today to tell him that all work-up including head imaging was negative. Upon the doctor’s recommendation, he decided to bring Melanie here.

**You are now preparing to go back to see the patient after you’ve staffed the case with your attending. The husband has left at this point. You would like to commit the patient for the following reasons:**

- **Pt is at imminent risk of harm to herself due to inability to care for self (not eating, not sleeping properly, not able to hold a job)**
- **Husband has expressed that he can no longer handle your behaviors at home, thus there is no other safe alternative**

4. Examinee Tasks:

1. Talk through the preparatory steps you would take before you see the patient.
2. See the patient and inform them that they are being involuntarily committed.
3. Exit the interview when you think it is appropriate to do so.

**EXAMINEE INSTRUCTIONS**

**Case Name:** Case #3

1. **Opening Scenario**

- Patient name: Jake Kirby
- Sex: Male
- Presents to: Local emergency psychiatry services
- Chief complaint: suicide attempt via overdose

1. **Vital Signs**

- Temperature: 98°F
- Blood Pressure: 115/76 mmHg
- Pulse: 76 bpm
- Respirations: 16 breaths per min

1. **Case**

You are the resident seeing Jake Kirby, a 20-year-old male who works as a waiter at Party Fowl. He is presenting to local psychiatry emergency services after being medically cleared at the main medical hospital for overdose on Benadryl, for evaluation of suicide attempt.

Per your chart review, at the medical hospital, he had presented as delirious and was admitted to the intensive care unit for vital sign abnormalities. His mental status improved to baseline over time and he was discharged to your current psychiatric facility after 5 days.

You go see Jake, and he is calm and cooperative on interview. He tells you that he was not intending to kill himself. He does state he had been having a rough time related to his mother’s health and increasing tension between him and his girlfriend. He states he took the Benadryl because he was having trouble going to sleep and did not realize he was taking too much. He did state he had been feeling depressed and called off from work for about two days to “take a break for my mental health.” Otherwise, he denies any issues with his ability to function at his job or engage in self-care at home. He thought of hanging himself a year ago but denies any other history of suicidal thoughts.

Jake is open to help for his mental health, but at an outpatient level. He adamantly refuses inpatient psychiatric admission, because his mom and other family members have been admitted before and from what he’s seen and heard, it is not a positive experience and “I’m not crazy like they are.” He wants to go home with his girlfriend, Jessica. He reluctantly gives consent for you to call her.

You call Jessica, who reveals a history of concerning behavior the weeks leading up to the event. She states “he’s a mess” – he has been staying in bed all day, neglecting self-care, and rarely leaving the house. He had called off work sick for the week. The day of his suicide attempt, he had sent a picture of a suicide note to Jessica, and she immediately left work to drive home to him.

Jessica endorses several recent stressors. She states Jake’s mother had been in the hospital multiple times for cardiac arrest secondary to drug overdose. His best friend completed suicide 3 years ago, and it is approaching his anniversary; Jessica notices that he has had suicidal behaviors every year around this time. For that reason, she had locked up all the pills in the house. However, Jake had revealed in his suicide note that for months, he had been asking for and collecting Benadryl tablets from her, with the intent of overdosing to end his life.

Jessica has been urging him to get mental health help, but he has “no-showed” to several of his appointments and instead been sleeping in, which led to eventual dismissal from the practice. She has recently mentioned to him that she plans to leave him if he continues to not seek/follow through with mental health care. She does not feel that she can manage him at home, given that she needs to go to work every day. She states his mother is not doing well mentally to have Jake live with her, and his dad lives in another state and has not been involved in his life recently.

**You are now preparing to go back to see the patient after you’ve called collateral and staffed the case with your attending. You would like to commit the patient for the following reasons:**

- **Concern for attempted suicide, other suicidal behaviors including premeditation of suicide attempt and preparatory actions, and poor insight into these behaviors that are concerning for imminent risk of self-harm**
- **Lack of self-care at home/functional decline also posing imminent risk of harm to self**
- **Girlfriend is not able to stay at home with him at all times, and family are not plausible safe options, thus there is no other safe alternative**

4. Examinee Tasks:

1. Talk through the preparatory steps you would take before you see the patient.
2. See the patient and inform them that they are being involuntarily committed.
3. Exit the interview when you think it is appropriate to do so.
