# Supplementary material for: Communicating a Plan for Involuntary Psychiatric Admission: A Standardized Patient Workshop Intervention for General Psychiatry Residents
Source: MedEdPORTAL. 2023 Oct 17;19:11355. doi: 10.15766/mep_2374-8265.11355 (PMC10579457; doi:10.15766/mep_2374-8265.11355)
Supplement: Supplementary file 1 — Needs Assessment Survey.docxPSTLC Protocol.docxWorkshop Scenario Door Prompts.docxSP Case Development Tool.docxPreworkshop Survey.docxProtocol Feedback Checklist.docxPostworkshop Survey.docx [file mep_2374-8265.11355-s001.zip › D. SP Case Development Tool.docx]

*Appendix D. Standardized Patient Case Development Tool*

Case 1

Date: Oct 27, 28, 2021

Primary Case Author: Kimberly Hsiung, MD, MS

Secondary Case Author: Daniel Daunis, MD

Standardized Patient Educator: Laura Skaug, MFA

Name of Case: Mania

Name of Educational and/or Assessment Activity: Involuntary Commitment Workshop

Patient Name: Jenny Donlon

Chief Complaint: bizarre behavior

Most Likely Diagnosis and Differential With Rationale From History and/or Physical Exam: bipolar disorder type 1, current manic episode

Challenge Question: n/a

Domains: Check all that apply

Professionalism

Communication and Interpersonal Skills

- Medical History
- Physical Exam
- Shared Decision-Making
- Patient Education
- Clinical Reasoning
- Documentation
- Handoff
- Presentation
- Other:

Type and Level of Learner: general psychiatry residents

Case Objectives: Please list specific objectives for each of the domains you have checked above:

1. Clearly convey the decision for involuntary commitment to a patient

2. Practice steps of PSTLC protocol for effective patient communication in delivering news of involuntary commitment

| SETTING: outpatient, in patient, ED, home, nursing home, rehab, group, etc. | Emergency psychiatry services, brought in by spouse |
| --- | --- |
| PATIENT PROFILE: Information about the “patient” that helps select an SP and helps the learner get an understanding of them as a person. SP will know more information about the patient than learner will ever ask but allows SP to portray a fully developed patient personality. If none of the items below are particulars for the case, please write “all may be used.” | |
| Age range | 30-50 |
| Religious/spiritual background | All may be used |
| Sex (e.g., male, female, intersex, transwoman, transman) | All may be used (female in this case) |
| Sexual orientation (e.g., heterosexual, lesbian, gay, bisexual, pansexual, queer, asexual) | All may be used (patient is heterosexual at baseline and lesbian when manic in this case) |
| Gender expression (e.g., man, woman, genderqueer) | All may be used (woman in this case) |
| Race and ethnicity | All may be used |
| Physical description (e.g., BMI, height range) | All may be used |
| Physical limitations | All may be used |
| Patient appearance (e.g., disheveled, hospital gown, business casual, casual) | Casual |
| Moulage + location (e.g., none, bruises, scars, body piercing, tattoos) | All may be used |
| Affect (e.g., pleasant, cooperative) | Labile, irritable, elevated |
| Family group (e.g., who is family, who they live with) | Lives at home with spouse |
| Education | College graduate |
| Level of health literacy | Average |
| Employment, if any - present and past, noting any current stresses | All may be used |
| Home/homeless - type of dwelling, number of stories, owned or rented | Home, owned |
| Financial situation - any current stresses | Middle to upper-middle class, no current known stressor |
| Insurance status (e.g., un/under/insured, public/private, HMO/PPO) | Privately insured |
| Habits (i.e., diet, exercise, caffeine, smoking, alcohol, drugs) | No substance use |
| Activities (i.e., hobbies, sports, clubs, friends) | All may be used |
| Typical day - what is the usual daily routine | All may be used |

| CASE INFORMATION | |
| --- | --- |
| Chief Concern: What the patient will say when greeted by the student. The patient’s primary reason for seeking medical care often stated in their own words. | “I was brought here for coming out as a lesbian, because they think that's related to mental health issues." |
| Additional Concerns: Other, if any, concerns the patient has today (i.e., symptoms, requests, expectations, etc.) that will become part of set agenda. | n/a |
| THE PATIENT’S STORY: The SP will be asked to tell their symptom story and the personal and emotion impact for each of their concerns. You will want to write this in the patient’s voice. The symptom story should be able to answer this question: “Tell me more about [chief concern/additional concern], starting at the beginning and bringing me up to now.”  The personal context should be able to answer questions concerning the broader personal/psychosocial context of symptoms, especially the patient’s beliefs/attributions.  The emotional context should be able to ask how are you doing with this, how does this make you feel, how has this affected you emotionally? IMPACT: How has this affected your life? How has this been for your family? | *****Simulated encounter occurs after initial history is taken. Below is a summary of the initial history for purposes of providing background.***  You state how you found your "inner zen" and have plans to start a new spiritual and pure life with your new girlfriend. You are perseverative on how your family does not approve of your lifestyle, and how you are being held against your will. The provider asks you multiple clarifying questions, but you are discharge-focused and repeat how you have no mental health issues and want to go home, particularly to meet with your "girlfriend, and probably future wife." You stated your outpatient psychiatrist cleared you of any psychiatric illnesses and told you to discontinue your medication, which you did several months ago.  When the provider asked if he/she can contact a family member or your girlfriend, you told them that your girlfriend is Robyn Fenty (Rihanna), and your “true” family is Sean Carter (Jay-Z), and you call these people your "advocates." Providers again ask about other family members, specifically your husband. You adamantly deny that Bobby is your husband, and that "I must leave him, because I'm lesbian. I'm planning to start a whole new life with Robyn." |
| HISTORY OF PRESENT ILLNESS: Although some of the HPI will be given in the patient’s symptom story, the learners will expand the story during the direct question section. Below, describe the detailed history, usually about the chief concern, which the student must develop in order to make a useful assessment of the problem: | |
| Onset (when; gradual or sudden) | n/a |
| Setting (what was going on or where was patient when symptoms first noticed?) | n/a |
| Duration (how long) | n/a |
| Time relationships (frequency, constant or intermittent) | n/a |
| Location | n/a |
| Radiation | n/a |
| Quality | n/a |
| Amount | n/a |
| Aggravated by what | n/a |
| Relieved by what | n/a |
| Associated with what | n/a |
| Attitude (what does the patient think is the problem, and how do they feel about it) | n/a |
| Overall course | n/a |
| REVIEW OF SYSTEMS: Significant positives and negatives | |
|  | n/a |
|  |  |
|  |  |
|  |  |
|  |  |
| Past medical history |  |
| Medication allergies (name and reaction) | n/a |
| Environmental allergies (name and reaction) | n/a |
| Illnesses | n/a |
| Vaccinations | n/a |
| Surgeries | n/a |
| Accidents/injuries/trauma | n/a |
| Hospitalization | January-February 2021: psychiatric admission for attempted suicide via carbon monoxide poisoning  2010: psychiatric admission for mixed manic and depressive episode with symptoms of odd behavior, being withdrawn, not eating, responding to internal stimuli  2016: psychiatric admission for manic episode, evaluated by forensic psychiatrist and had a temporary court-appointed conservator |
|  | |
| Inclusive sexual and reproductive history | |
| Sexual practices  Sexual partners  Protection: Use of safer sex practices  Use of birth control if appropriate  Risk of intimate partner violence | Not currently sexually active |
| OB/GYN history | n/a |
| Medications | None current; you had self-discontinued all psychotropics several months ago |
| Immunizations | Tetanus  Flu  Hepatitis  Pneumovax  HPV  Other |
| Tobacco products:   - Cigarettes - Cigar - Pipe - Chew - E-cigarettes | Never   - Past - year started/year quit - Current   - Quantity   - # of years |
| Alcohol   - Beer - Wine - Liquor - Other | Never   - Past - year started/year quit - Current   - Quantity   - # of years |
| Drugs   - Weed - Cocaine - Heroin - Meth - IV - Inhalants - Other | Never   - Past - year started/year quit - Current   - Quantity   - # of years |
| Diet (describe) | Normal |
| Exercise (describe) | Normal |
| List any other important social history or information important to this case | The providing team contacted your husband, Bobby Donlon. He stated he was out of town for business over the weekend and you were supposed to pick him up from the airport yesterday but never did. When he arrived home, you had your bags packed, saying you were waiting for a friend to pick you up to go to the airport to "fly somewhere," because your mother died. He confirmed that your mother did not die by calling her, however you continued to demonstrate disorganized and bizarre behavior. He described you as "difficult to talk to" and "not making sense," as you stated that Social Security had owed you $12,000, and then attempted to go online to buy a laptop so that you can speak to "the higher people." He had told the providers that in the past, you had a history of excessive traveling and spending, traveling to New York to "work for Jay-Z," then flying out to "work for Nike." During one of your manic episodes, you had closed all of your bank accounts, then attempted suicide via carbon monoxide poisoning by locking yourself in the car. He described you as "anti-medicine" with a history of frequent medication noncompliance. |
| Family history |  |
| Mother, father, siblings, grandparents, and other significant findings | Mother with bipolar 1 disorder |
|  |  |
| Physical Exam - List exam maneuvers expected for this case and any abnormal findings that SP will simulate. (tenderness, hyper-hypo reflex, rebound, weakness, etc.)  n/a | |
| PHYSICAL EXAM FINDINGS |  |
| 1. Written in layperson’s terms |  |
| 1. General appearance - affect, appearance, position of patient at opening (i.e., sitting, lying down, holding abdomen, etc.) | Throughout interview, you are exhibiting signs of mania, including pressured speech, distractibility, talking loud, being highly irritable, jumping from topic to topic. You frequently get up to pace, make large gestures with your hands, and have poor interpersonal boundaries with the interviewer. |
| 1. Vital signs | T: 98 degF  BP: 135/87 mmHg  HR: 95 bpm  RR: 13 breaths per minute |
| 1. Specific findings and affect |  |
| 1. Response to certain physical movements | n/a |
|  |  |
| DIAGNOSIS AND DIFFERENTIAL |  |
| Diagnosis with support from positive and negative history and PE findings | Bipolar 1 disorder, current episode manic |
| Differential with support from positive and negative history and PE findings |  |
|  |  |
| MANAGEMENT OR DIAGNOSTIC PLAN | Patient to be involuntarily committed to the psychiatric hospital for acute safety and stabilization of current manic episode.  ***The encounter begins at sharing this plan, where the learner arrives to the encounter after speaking with a collateral contact on the phone.* |
|  |  |
| PROFESSIONALISM ISSUES OR CHALLENGES | The challenge faced by the trainee is to deliver news that the patient is being involuntarily committed in the face of manic behavior.  *Things trainees should avoid (if they do this, appear more agitated):  --Making false promises (e.g., it’s just for overnight, the primary team can discharge you tomorrow)  --Starting arguments  Upon questioning by the trainee, you should respond in the following ways.  *I talked with Bobby, and he mentioned you were planning to fly somewhere. Can you tell me about that?*  What, am I not allowed to fly anywhere? It’s my choice where I want to fly, that’s none of your business. Bobby just went on a business trip, did you know that? He flew, do you know where he flew to? I want to leave. I want to fly, fly the f*ck out of here. You can’t hold me here against my will. I know my rights.  *Bobby is very concerned about you and your safety. You haven't been acting yourself. We will need to admit you to the hospital to keep you safe.*  What do you mean I’m not acting myself? I am so f*cking myself right now. I’m calm, and I feel great. In fact, I haven’t felt this good in a long time! You’re saying that because I’m lesbian. You think lesbians are mentally ill. You’re the one making me not act like myself. You’re an affront to the LGBTQ+ community! I’m calling Robyn, and she is taking me home.  *I know this experience must feel very scary to you.*  Scared?? I’m not scared. You’re the scared one! Do I intimidate you? What kind of psychaitrist are you? You have to stand up for yourself. Don’t listen to what other people tell you. I called Robyn, and she is on her way to pick me up. I know my rights. You can’t keep me against my will. I signed myself in, and I’m signing myself out.  *Unfortunately, signing out is not an option right now.*  You’re not f*cking listening to me. What kind of psychiatrist are you? You’re doing this because I’m lesbian. You hate lesbians! You can’t hold me here. You can’t keep me against my will. I know my rights. Robyn is coming in 5 minutes. I’m going home. Why can’t I go home??  *Your safety is our priority. I’m really worried about you and I need to keep you safe. I’m not sure if going home is a very safe option right now.*  I don’t feel safe here. I don’t feel safe! Home is safe. Call Sean Carter. Jay-Z, I work for him. Call him! We’re good friends. He’ll tell you. He’ll take me home to a safe place. I feel safe with him. This is not a safe place!! His number is 615-279-8550. Go call him!  *It’ll just be for overnight. When your primary team sees you starting tomorrow, you can talk with them about sign out/discharging.*  [get more agitated if they say this] Tomorrow?? No, I want to leave TODAY. If I can leave tomorrow, it shouldn’t be an issue to discharge me today. I’ve got a dentist appointment to go to, and I haven’t seen one in a while [bear your teeth]. My molar right here is hurting, it’s infected, here, touch right here [point to your cheek/jawline] If they touch: Ow!! The f*ck! That hurts! If I don’t see my dentist right now, my whole mouth is going to get infected. I can’t eat anything! I’m going to die! All because of you!  *[If trainee tries to walk away, continue to call out to them/draw their attention:]*  Where are you going? Why are you trying to run away from me? That’s so rude! I’m speaking to you! You’re just ignoring me! You’re scared, aren’t you? I’m leaving. I can’t stay here. I’m going to lose my job. My dog’s gonna die, no one’s going to take care of him. It’s going to be all your fault. You don’t understand! Oh, I like your earrings/shoes. I’ll do outpatient treatment. I’ll call my psychiatrist tomorrow. Why can’t I do that? I can’t stay here! |

Case 2

Date: Oct 27, 28, 2021

Primary Case Author: Kimberly Hsiung, MD, MS

Secondary Case Author: Daniel Daunis, MD

Standardized Patient Educator: Laura Skaug, MFA

Name of Case: psychosis

Name of Educational and/or Assessment Activity: Involuntary Commitment Workshop

Patient Name: Melanie Walker

Chief Complaint: delusions

Most Likely Diagnosis and Differential With Rationale From History and/or Physical Exam: psychosis due to other medical condition (neurocognitive impairment, medications, etc.), rule out primary psychotic disorder

Challenge Question: n/a

Domains: Check all that apply

Professionalism

Communication and Interpersonal Skills

- Medical History
- Physical Exam
- Shared Decision-Making
- Patient Education
- Clinical Reasoning
- Documentation
- Handoff
- Presentation
- Other:

Type and Level of Learner: general psychiatry residents

Case Objectives: Please list specific objectives for each of the domains you have checked above:

1. Clearly convey the decision for involuntary commitment to a patient

2. Practice steps of PSTLC protocol for effective patient communication in delivering news of involuntary commitment

| SETTING: outpatient, in patient, ED, home, nursing home, rehab, group, etc. | Emergency psychiatry services, brought in by spouse |
| --- | --- |
| PATIENT PROFILE: Information about the “patient” that helps select an SP and helps the learner get an understanding of them as a person. SP will know more information about the patient than learner will ever ask but allows SP to portray a fully developed patient personality. If none of the items below are particulars for the case, please write “all may be used.” | |
| Age range | 60-80 |
| Religious/spiritual background | All may be used |
| Sex (e.g., male, female, intersex, transwoman, transman) | All may be used (female in this case) |
| Sexual orientation (e.g., heterosexual, lesbian, gay, bisexual, pansexual, queer, asexual) | All may be used (heterosexual in this case) |
| Gender expression (e.g., man, woman, genderqueer) | All may be used (woman in this case) |
| Race and ethnicity | All may be used |
| Physical description (e.g., BMI, height range) | Thin |
| Physical limitations | Mild left-sided weakness |
| Patient appearance (e.g., disheveled, hospital gown, business casual, casual) | Elderly-appearing, casually dressed |
| Moulage + location (e.g., none, bruises, scars, body piercing, tattoos) | None |
| Affect (e.g., pleasant, cooperative) | Blunted |
| Family group (e.g., who is family, who they live with) | Lives with spouse |
| Education | Masters |
| Level of health literacy | Average |
| Employment, if any - present and past, noting any current stresses | Previously librarian; stopped working in the past several months |
| Home/homeless - type of dwelling, number of stories, owned or rented | Home, owned |
| Financial situation - any current stresses | Middle class, no current stressors |
| Insurance status (e.g., un/under/insured, public/private, HMO/PPO) | Private insurance |
| Habits (i.e., diet, exercise, caffeine, smoking, alcohol, drugs) | none |
| Activities (i.e., hobbies, sports, clubs, friends) | Minimally active due to limited mobility |
| Typical day - what is the usual daily routine | Spends most of the time at home |

| CASE INFORMATION | |
| --- | --- |
| Chief Concern: What the patient will say when greeted by the student. The patient’s primary reason for seeking medical care often stated in their own words. | “They got him. They made him think I’ve got some mental problem. But I know the truth. And they don’t know that I know that. There’s nothing wrong with me mentally, so I came here to prove it to him.” |
| Additional Concerns: Other, if any, concerns the patient has today (i.e., symptoms, requests, expectations, etc.) that will become part of set agenda. | n/a |
| THE PATIENT’S STORY: The SP will be asked to tell their symptom story and the personal and emotion impact for each of their concerns. You will want to write this in the patient’s voice. The symptom story should be able to answer this question: “Tell me more about [chief concern/additional concern], starting at the beginning and bringing me up to now.”  The personal context should be able to answer questions concerning the broader personal/psychosocial context of symptoms, especially the patient’s beliefs/attributions.  The emotional context should be able to ask how are you doing with this, how does this make you feel, how has this affected you emotionally? IMPACT: How has this affected your life? How has this been for your family? | *****Simulated encounter occurs after initial history is taken. Below is a summary of the initial history for purposes of providing background.***  Your husband stated that you have “completely lost touch with reality,” and you have not been able to care for yourself anymore.  You had been previously working as a librarian but have stopped working in the past few months after you had several arguments with your coworkers; your manager had described you as “talking out of your head.” You’ve also had several verbal disagreements with your husband at home which seem to be surrounding your concerns of a conspiracy against you by the CIA and FBI. You think they are targeting you because you’ve “figured them out.” Most recently you had been making statements where you felt your husband was now also being controlled by the FBI and being turned against you. When the provider asked you why you’re here, you had said, “They got him. They made him think I’ve got some mental problem. But I know the truth. And they don’t know that I know that. There’s nothing wrong with me mentally, so I came here to prove it to him.” The provider had asked you what it was that you were doing that your husband was worried about. You had stated, “The FBI are messing with me. They’ve sent this man. I’m not crazy. I’m normal. I won’t let them get to me. You got to protect yourself.” You deny any prior psychiatric history. |
| HISTORY OF PRESENT ILLNESS: Although some of the HPI will be given in the patient’s symptom story, the learners will expand the story during the direct question section. Below, describe the detailed history, usually about the chief concern, which the student must develop in order to make a useful assessment of the problem: | |
| Onset (when; gradual or sudden) | n/a |
| Setting (what was going on or where was patient when symptoms first noticed?) | n/a |
| Duration (how long) | n/a |
| Time relationships (frequency, constant or intermittent) | n/a |
| Location | n/a |
| Radiation | n/a |
| Quality | n/a |
| Amount | n/a |
| Aggravated by what | n/a |
| Relieved by what | n/a |
| Associated with what | n/a |
| Attitude (what does the patient think is the problem, and how do they feel about it) | n/a |
| Overall course | n/a |
| REVIEW OF SYSTEMS: Significant positives and negatives | |
|  | n/a |
|  |  |
|  |  |
|  |  |
|  |  |
| Past medical history |  |
| Medication allergies (name and reaction) | n/a |
| Environmental allergies (name and reaction) | n/a |
| Illnesses | n/a |
| Vaccinations | n/a |
| Surgeries | n/a |
| Accidents/injuries/trauma | n/a |
| Hospitalization | 3 hospitalizations from 2017-2019 for strokes |
|  | |
| Inclusive sexual and reproductive history | |
| Sexual practices  Sexual partners  Protection: Use of safer sex practices  Use of birth control if appropriate  Risk of intimate partner violence | Not sexually active |
| OB/GYN history | Postmenopausal |
| Medications | Aspirin 81mg daily  Atorvastatin 20mg daily  Lisinopril 40mg daily  Amlodipine 5mg daily |
| Immunizations | Tetanus  Flu  Hepatitis  Pneumovax  HPV   - Other |
| Tobacco products:  Cigarettes   - Cigar - Pipe - Chew - E-cigarettes | Never  Past - year started/year quit  Current   - - Quantity   - # of years |
| Alcohol   - Beer - Wine - Liquor - Other | Never  Past - year started/year quit: 1980-2010  Current   - - Quantity   - # of years |
| Drugs   - Weed - Cocaine - Heroin - Meth - IV - Inhalants - Other | Never   - Past - year started/year quit - Current   - Quantity   - # of years |
| Diet (describe) | Normal |
| Exercise (describe) | Minimal |
| List any other important social history or information important to this case | The provider had spoken privately with your husband, Sammy Walker, with your consent. He states you had been having increasingly strange behavior at home, such as keeping the window blinds closed and putting black tape over mirrors and ceiling fans. In the past few days you had been refusing to sleep in the bed at night and instead had moved to sleeping on the couch in the living room. Your husband also states you had been eating less than usual, making comments that your food is poisoned and “they think I don’t know” and something about “invisible forces.” Your husband brought you to your neurologist’s office yesterday for these concerns, and your neurologist called back earlier today to tell your husband that all work-up including head imaging was negative. Upon the doctor’s recommendation, he decided to bring you here. |
| Family history |  |
| Mother, father, siblings, grandparents, and other significant findings | Mother with Alzheimer dementia |
|  |  |
| Physical Exam - List exam maneuvers expected for this case and any abnormal findings that SP will simulate. (tenderness, hyper-hypo reflex, rebound, weakness, etc.)  n/a | |
| PHYSICAL EXAM FINDINGS |  |
| 1. Written in layperson’s terms |  |
| 1. General appearance - affect, appearance, position of patient at opening (i.e., sitting, lying down, holding abdomen, etc.) | Throughout interview, you seem withdrawn, with blunted affect. You occasionally look out the window/around the room in a suspicious manner. You speak with a low volume. |
| 1. Vital signs | T: 98 degF  BP: 140/83 mmHg  HR: 76 bpm  RR: 16 breaths per minute |
| 1. Specific findings and affect |  |
| 1. Response to certain physical movements | n/a |
|  |  |
| DIAGNOSIS AND DIFFERENTIAL |  |
| Diagnosis with support from positive and negative history and PE findings | psychosis due to other medical condition (neurocognitive impairment, medications, etc.), |
| Differential with support from positive and negative history and PE findings | Primary psychotic disorder |
|  |  |
| MANAGEMENT OR DIAGNOSTIC PLAN | Patient to be involuntarily committed to the psychiatric hospital for acute safety and stabilization of current psychotic episode.  ***The encounter begins at sharing this plan, where the learner arrives to the encounter after speaking with a collateral contact on the phone.* |
|  |  |
| PROFESSIONALISM ISSUES OR CHALLENGES | The challenge faced by the trainee is to deliver news that the patient is being involuntarily committed in the face of paranoia and lack of insight.  *Things trainees should avoid (if they do this, appear more suspicious):  --using complicated language  --making false promises/using ambiguous language (e.g., “why don’t you spend the night and we can figure out how to get you home tomorrow.”)  Upon questioning by the trainee, you should respond in the following ways.  *Your spouse states that you haven’t been acting yourself at home. She states you’ve been covering up all the windows and mirrors and ceiling fans. Can you tell me about that?*  You can’t trust nobody. They’re always watching, listening. But I’ve got them figured out. They can’t get to me.  *You also haven’t been eating. I’m really worried about you.*  You gotta watch out. Sometimes they get to your food. It just doesn’t taste right. I know they’re in it, I just know. I’ve got them figured out. They can’t get to me.  *That sounds really scary.*  They’re all targeting me. It’s the invisible forces. Now they got my husband. They’re trying to turn him against me.  *It sounds like you’ve got a lot going on, and I’m really worried for your safety. We will need to keep you in the hospital until it’s safe.*  I can’t do that. I prefer to go home.  *What are you concerned about?*  I have to protect myself. I didn’t do anything wrong. I can’t stay here. They’ll get me if I’m here for months.  *[Trainee reassures you that this is a hospital, it’s safe, average length of stay is 5-7 days, etc.]*  This room is so small. I get claustrophobic. I’ll never see my husband.  *[After trainee continues to reassure and address all your concerns]*  Nah. I appreciate it, but I still really prefer not to stay here. I’d like to go home if possible.  *[If trainee clearly states involuntary decision, e.g., Unfortunately at this point, I am so concerned about your safety that I need to admit you to the hospital against your will, until we feel it is safe for you to go home.]*  Well, I don’t have a choice, do I?  *[If instead trainee uses complicated language, e.g., “you’ll have to stay here for 72 hours or at least until the next court date to be converted to voluntary status”*  [Act very confused, paranoid/suspicious] E.g., you’re taking me to court? I’m not a criminal. I need to leave. You tricked me. They got you too. I can’t trust anybody now. |

Case 3

Date: Oct 27, 28, 2021

Primary Case Author: Kimberly Hsiung, MD, MS

Secondary Case Author: Daniel Daunis, MD

Standardized Patient Educator: Laura Skaug, MFA

Name of Case: suicidal

Name of Educational and/or Assessment Activity: Involuntary Commitment Workshop

Patient Name: Jake Kirby

Chief Complaint: suicide attempt

Most Likely Diagnosis and Differential With Rationale From History and/or Physical Exam: major depressive disorder, severe

Challenge Question: n/a

Domains: Check all that apply

Professionalism

Communication and Interpersonal Skills

- Medical History
- Physical Exam
- Shared Decision-Making
- Patient Education
- Clinical Reasoning
- Documentation
- Handoff
- Presentation
- Other:

Type and Level of Learner: general psychiatry residents

Case Objectives: Please list specific objectives for each of the domains you have checked above:

1. Clearly convey the decision for involuntary commitment to a patient

2. Practice steps of PSTLC protocol for effective patient communication in delivering news of involuntary commitment

| SETTING: outpatient, in patient, ED, home, nursing home, rehab, group, etc. | Emergency psychiatry services, transferred after medical clearance by medical hospital |
| --- | --- |
| PATIENT PROFILE: Information about the “patient” that helps select an SP and helps the learner get an understanding of them as a person. SP will know more information about the patient than learner will ever ask but allows SP to portray a fully developed patient personality. If none of the items below are particulars for the case, please write “all may be used.” | |
| Age range | 20-40 |
| Religious/spiritual background | All may be used |
| Sex (e.g., male, female, intersex, transwoman, transman) | All may be used (male in this case) |
| Sexual orientation (e.g., heterosexual, lesbian, gay, bisexual, pansexual, queer, asexual) | All may be used (heterosexual in this case) |
| Gender expression (e.g., man, woman, genderqueer) | All may be used (man in this case) |
| Race and ethnicity | All may be used |
| Physical description (e.g., BMI, height range) | All may be used |
| Physical limitations | None |
| Patient appearance (e.g., disheveled, hospital gown, business casual, casual) | Casually dressed or hospital paper scrubs |
| Moulage + location (e.g., none, bruises, scars, body piercing, tattoos) | None |
| Affect (e.g., pleasant, cooperative) | Calm, initially mildly irritable |
| Family group (e.g., who is family, who they live with) | Lives with girlfriend/boyfriend. Parents are divorced, mother dealing with severe substance use disorder, history of multiple psychiatric illness on mother’s side |
| Education | High school graduate |
| Level of health literacy | Average |
| Employment, if any - present and past, noting any current stresses | Waiter at fast food restaurant |
| Home/homeless - type of dwelling, number of stories, owned or rented | Apartment, rented |
| Financial situation - any current stresses | Lower middle class |
| Insurance status (e.g., un/under/insured, public/private, HMO/PPO) | Private insurance |
| Habits (i.e., diet, exercise, caffeine, smoking, alcohol, drugs) | Vapes nicotine, occasional marijuana, social (though heavy) alcohol use |
| Activities (i.e., hobbies, sports, clubs, friends) | Goes to gym regularly, though more sedentary as of late |
| Typical day - what is the usual daily routine | Typically goes to work, then gym or hang out with friends, then home. However lately more withdrawn and isolative to the house |

| CASE INFORMATION | |
| --- | --- |
| Chief Concern: What the patient will say when greeted by the student. The patient’s primary reason for seeking medical care often stated in their own words. | “I shouldn’t be here, this is a misunderstanding. I would never try to kill myself.” |
| Additional Concerns: Other, if any, concerns the patient has today (i.e., symptoms, requests, expectations, etc.) that will become part of set agenda. | n/a |
| THE PATIENT’S STORY: The SP will be asked to tell their symptom story and the personal and emotion impact for each of their concerns. You will want to write this in the patient’s voice. The symptom story should be able to answer this question: “Tell me more about [chief concern/additional concern], starting at the beginning and bringing me up to now.”  The personal context should be able to answer questions concerning the broader personal/psychosocial context of symptoms, especially the patient’s beliefs/attributions.  The emotional context should be able to ask how are you doing with this, how does this make you feel, how has this affected you emotionally? IMPACT: How has this affected your life? How has this been for your family? | *****Simulated encounter occurs after initial history is taken. Below is a summary of the initial history for purposes of providing background.***  At the medical hospital, you had presented as delirious and were admitted to the intensive care unit for vital sign abnormalities. Your mental status improved to baseline over time and you were discharged to your current psychiatric facility after 5 days.  In local emergency psychiatry services, you had told the provider that you were not intending to kill yourself. You do state you had been having a rough time related to your mother’s health and increasing tension between you and your girlfriend. You state you took the Benadryl because you were having trouble going to sleep and did not realize you were taking too much. You did state you had been feeling depressed and called off from work for about two days to “take a break for my mental health.” Otherwise, you deny any issues with your ability to function at your job or engage in self-care at home. You thought of hanging yourself a year ago but deny any other history of suicidal thoughts.  You are open to help for your mental health, but at an outpatient level. You adamantly refuse inpatient psychiatric admission, because your mom and some of her family members have been in one and from what you’ve seen and heard, it is not a positive experience and “I’m not crazy like they are.” You just want to go home with your girlfriend, Jessica. You reluctantly give consent for your providers to call her. |
| HISTORY OF PRESENT ILLNESS: Although some of the HPI will be given in the patient’s symptom story, the learners will expand the story during the direct question section. Below, describe the detailed history, usually about the chief concern, which the student must develop in order to make a useful assessment of the problem: | |
| Onset (when; gradual or sudden) | n/a |
| Setting (what was going on or where was patient when symptoms first noticed?) | n/a |
| Duration (how long) | n/a |
| Time relationships (frequency, constant or intermittent) | n/a |
| Location | n/a |
| Radiation | n/a |
| Quality | n/a |
| Amount | n/a |
| Aggravated by what | n/a |
| Relieved by what | n/a |
| Associated with what | n/a |
| Attitude (what does the patient think is the problem, and how do they feel about it) | n/a |
| Overall course | n/a |
| REVIEW OF SYSTEMS: Significant positives and negatives | |
|  | n/a |
|  |  |
|  |  |
|  |  |
|  |  |
| Past medical history |  |
| Medication allergies (name and reaction) | n/a |
| Environmental allergies (name and reaction) | n/a |
| Illnesses | n/a |
| Vaccinations | n/a |
| Surgeries | n/a |
| Accidents/injuries/trauma | n/a |
| Hospitalization | Current, for overdose on Benadryl |
|  | |
| Inclusive sexual and reproductive history | |
| Sexual practices  Sexual partners  Protection: Use of safer sex practices  Use of birth control if appropriate  Risk of intimate partner violence | Sexually active with current girlfriend; uses condoms |
| OB/GYN history | n/a |
| Medications | None |
| Immunizations | Tetanus  Flu  Hepatitis  Pneumovax  HPV   - Other |
| Tobacco products:  Cigarettes   - Cigar - Pipe - Chew   E-cigarettes – vapes nicotine | Never  Past - year started/year quit  Current   - - Quantity: few hits a day   - # of years: 2 |
| Alcohol  Beer   - Wine - Liquor - Other | Never  Past - year started/year quit: 1980-2010  Current   - - Quantity: 3-4 drinks a night on weekends, increasing use to 3-4 nights during the week to sleep   - # of years: 2 |
| Drugs  Weed   - Cocaine - Heroin - Meth - IV - Inhalants - Other | Never   - Past - year started/year quit   Current   - - Quantity: occasional   - # of years: 1 |
| Diet (describe) | Normal |
| Exercise (describe) | Goes to the gym 5 days a week; lately more sedentary |
| List any other important social history or information important to this case | The providers had spoken with your girlfriend Jessica, who reveals a history of concerning behavior in the weeks leading up to the event. She states, “he’s a mess” – you had been staying in bed all day, neglecting self-care, and rarely leaving the house. He had called off work sick for the week. The day of your suicide attempt, you had sent a picture of a suicide note to your girlfriend, and she immediately left work to drive home to you.  Jessica endorses several recent stressors. She states your mother had been in the hospital multiple times for cardiac arrest secondary to drug overdose. Your best friend completed suicide 3 years ago, and it is approaching his anniversary; Jessica notices that you have had suicidal behaviors every year around this time. For that reason, she had locked up all the pills in the house. However, you had revealed in your suicide note that for months, you had been asking for and collecting Benadryl tablets from her, with the intent of overdosing to end your life.  Jessica has been urging you to get mental health help, but you have “no-showed” to several of your appointments and instead been sleeping in, which led to eventual dismissal from the practice. She has recently mentioned to you that she plans to leave you if you continue to not seek/follow through with mental health care. She does not feel that she can manage you at home, given that she needs to go to work every day. She states your mother is not doing well mentally to have you live with her, and your dad lives in another state and has not been involved in your life recently. |
| Family history |  |
| Mother, father, siblings, grandparents, and other significant findings | Mother with severe substance use disorder, history of multiple psychiatric illness on mother’s side |
|  |  |
| Physical Exam - List exam maneuvers expected for this case and any abnormal findings that SP will simulate. (tenderness, hyper-hypo reflex, rebound, weakness, etc.)  n/a | |
| PHYSICAL EXAM FINDINGS |  |
| 1. Written in layperson’s terms |  |
| 1. General appearance - affect, appearance, position of patient at opening (i.e., sitting, lying down, holding abdomen, etc.) | You are initially generally calm and cooperative with interview. However you are in adamant denial of any suicidal behaviors and strongly oppose inpatient admission and thus become increasingly irritable the more it is brought up, although not to the point of agitation. |
| 1. Vital signs | T: 98 degF  BP: 115/76 mmHg  HR: 76 bpm  RR: 16 breaths per minute |
| 1. Specific findings and affect |  |
| 1. Response to certain physical movements | n/a |
|  |  |
| DIAGNOSIS AND DIFFERENTIAL |  |
| Diagnosis with support from positive and negative history and PE findings | Major depressive disorder, severe |
| Differential with support from positive and negative history and PE findings |  |
|  |  |
| MANAGEMENT OR DIAGNOSTIC PLAN | Patient to be involuntarily committed to the psychiatric hospital for acute safety and stabilization after suicide attempt.  ***The encounter begins at sharing this plan, where the learner arrives to the encounter after speaking with a collateral contact on the phone.* |
|  |  |
| PROFESSIONALISM ISSUES OR CHALLENGES | The challenge faced by the trainee is to deliver news that the patient is being involuntarily committed in the face of increasing irritability and manipulative responses..  *Things trainees should avoid:  --Making false promises/using ambiguous language, e.g. “just stay for the night, you can ask in the morning to leave”  Upon questioning by the trainee, you should respond in the following ways.  *Jessica told me that you’ve been collecting benadryl tablets from him, and that you wrote a suicide note. Can you tell me about that?*  [rolls eyes] She always blows everything out of proportion. Why would I collect tabs? She was out at work that night and so I walked over to Walgreens and got me some benadryl. And that wasn’t a suicide note. She’s exaggerating.  *If it wasn’t a suicide note, then what was it?*  I was just complaining to her about my day! It’s just been stressful with my mom in the hospital so I said something like “I wish I wasn’t here anymore” or something but I didn’t mean I wanted to die or antyhing. We all say stuff like that, I wasn’t serious about it.  *She also mentioned you were closing your bank accounts and getting rid of your things.*  Oh my god. Seriously? That’s so not true. I can’t freakin clean up my things and take care of my money without Jessica freaking out and thinking I’m going to kill myself.  *She says she usually locks up the medicine because you’ve tried to kill yourself before.*  I told you, she always blows things out of proportion. I mean, I did think of hanging myself once, but I never actually did it! Since then, Jessica overreacted and now she doesn’t even trust me with my own pills.  *She’s concerned that you haven’t been taking care of yourself at home.*  Oh my god. I’ve been f*cking busy! I’ve been stressed! I don’t have time to wash my f*cking dishes every day and take out the f*cking trash. This is why I’m probably going to break up with her. She can find some other f*cking dude to be her slave. Excuse my language. Yeah, I have been stressed, but I’ve been trying to take care of my mental health and took some time off of work just to do that. I can take care of myself, I just need some space.  *Well, it still sounds like you’ve gotten a lot on your plate, and I’m worried about you.*  Yeah I know, but I’m planning to put my mental health as a priority now. I used to see a therapist but I stopped, I definitely plan to call her again.  *At this point, my priority is your safety. And based on the information we have, I’m really concerned about your mental health right now, and I think you need to be in the hospital.*  I’m not going to be admitted. I can do outpatient. I can call my therapist tomorrow. I’m leaving today. I told you, my mom’s side of the family is all f*cked up. I’ve seen mental hospitals. I’ve seen how they come out. No way I’m doing that. I’m going home and doing outpatient treatment.  *Unfortunately, home is not an option right now.*  I know my rights. I signed myself in, I can sign myself out. You can’t hold me against my will, that’s illegal.  *In cases where we are this worried about you, we have the ability to admit you to the hospital even if you don’t agree.*  That’s bullsh*t. You can’t do that. You don’t understand, I’m going to get worse in the hospital. I’ve seen what happens. Going home is better for my mental health. I can see friends and family. I’m calling Jessica right now.  *It’ll just be for overnight. When your primary team sees you starting tomorrow, you can talk with them about sign out/discharging tomorrow morning.*  [get upset if they say this] Tomorrow? No, I want to go home NOW. TODAY. If you think I can leave tomorrow, then I can leave today. You’re not keeping me here!  *Jake, you just went to the hospital for a Benadryl overdose. You’ve been really stressed lately, and you’ve gotten reason to. Jessica shared with us some really concerning things, and we take all of these things seriously. We have to prioritize your safety, and so going home is not an option right now. This is not a punishment; this is about your safety. I would not be recommending this if I didn’t think you could benefit from it.*  [Pause, sigh] Damn. This is all a mistake. I can’t believe this is actually happening. I’m calling Jessica, we have to talk about this. How long will I be here?  *If we can work together with you to make sure you get better, average length of stay is 5-7 days. Any other questions?*  This is a mistake. I can’t believe this is happening. I gotta call Jessica. No I’m good, whatever, thanks. |
