# Supplementary material for: Communicating a Plan for Involuntary Psychiatric Admission: A Standardized Patient Workshop Intervention for General Psychiatry Residents
Source: MedEdPORTAL. 2023 Oct 17;19:11355. doi: 10.15766/mep_2374-8265.11355 (PMC10579457; doi:10.15766/mep_2374-8265.11355)
Supplement: Supplementary file 1 — Needs Assessment Survey.docxPSTLC Protocol.docxWorkshop Scenario Door Prompts.docxSP Case Development Tool.docxPreworkshop Survey.docxProtocol Feedback Checklist.docxPostworkshop Survey.docx [file mep_2374-8265.11355-s001.zip › E. Preworkshop Survey.docx]

Appendix E. Preworkshop Survey

1. Year: [ ] PGY-1 [ ] PGY-2 [ ] PGY-3 [ ] PGY-4
2. Gender/sex: [ ] Male [ ] Female [ ] Other
3. How comfortable are you with your ability to deliver news to a patient that they are being involuntarily committed to VPH?

[ ] Not at all comfortable

[ ] Somewhat comfortable

[ ] Moderately comfortable

[ ] Very comfortable

[ ] Extremely comfortable

1. After learning the protocol steps discussed in last week’s didactic, how much do you intend to change your approach to involuntary commitment conversations going forward?

[ ] I intend to significantly change my approach

[ ] I intend to make some changes to my approach

[ ] I intend to make some tweaks to my approach

[ ] I intend to not change anything to my approach
