# Supplementary material for: Communicating a Plan for Involuntary Psychiatric Admission: A Standardized Patient Workshop Intervention for General Psychiatry Residents
Source: MedEdPORTAL. 2023 Oct 17;19:11355. doi: 10.15766/mep_2374-8265.11355 (PMC10579457; doi:10.15766/mep_2374-8265.11355)
Supplement: Supplementary file 1 — Needs Assessment Survey.docxPSTLC Protocol.docxWorkshop Scenario Door Prompts.docxSP Case Development Tool.docxPreworkshop Survey.docxProtocol Feedback Checklist.docxPostworkshop Survey.docx [file mep_2374-8265.11355-s001.zip › F. Protocol Feedback Checklist.docx]

Involuntary Commitment Workshop

Appendix F. P.S.TLC Protocol Feedback Checklist

1. PREP and safety

state whether or not they order prns, specify location of conversation

Comments:

1. SUMMARIZE concerns

state concerns

state rationale (e.g., “I’m worried that X will happen if you go home.”)

express that they are coming from a place of concern and desire to help

Overall comments:

1. Be TRANSPARENT and state involuntary commitment decision

state decision with transparency

no ambiguity/ambivalence

Overall comments:

1. LISTEN, empathize, re-emphasize decision

empathic listening

appropriate use of empathic statements

patient concerns are heard

re-state decision

Overall comments:

1. Post-conversation COMMUNICATION

state what they will communicate and to whom

Overall comments:
