# Supplementary material for: Communicating a Plan for Involuntary Psychiatric Admission: A Standardized Patient Workshop Intervention for General Psychiatry Residents
Source: MedEdPORTAL. 2023 Oct 17;19:11355. doi: 10.15766/mep_2374-8265.11355 (PMC10579457; doi:10.15766/mep_2374-8265.11355)
Supplement: Supplementary file 1 — Needs Assessment Survey.docxPSTLC Protocol.docxWorkshop Scenario Door Prompts.docxSP Case Development Tool.docxPreworkshop Survey.docxProtocol Feedback Checklist.docxPostworkshop Survey.docx [file mep_2374-8265.11355-s001.zip › _Educational Summary Report_11355.pdf]

MedEdPORTAL® The AAMC Journal of Teaching and Learning Resources

Original Publication Open Access

Communicating a Plan for Involuntary Psychiatric Admission: A Standardized Patient Workshop Intervention for General Psychiatry Residents

Kimberly Hsiung, MD, MS*, Laura Skaug, MFA, Daniel Daunis, MD

*Corresponding author: hsiung.kimberly.s@gmail.com

Abstract

Introduction: One important and often difficult act of communication common in psychiatry is communication regarding the need for involuntarily commitment for psychiatric treatment. Thus, we designed an educational workshop for psychiatry residents on how to communicate the plan for involuntarily commitment to a psychiatric hospital. **Methods:** Using faculty expertise, we created a protocol to guide trainees on how to structure conversations around involuntary commitment. Residents first attended a didactic on the protocol, followed by a 1-hour workshop with standardized patients (SPs) 1 week later. The workshop consisted of three 14-minute simulated scenarios with the SP with debriefing. Trainees filled out pre- and postworkshop surveys. **Results:** Fifteen and 12 residents completed the pre- and postworkshop surveys, respectively. Residents' perceived comfort level in their ability to deliver involuntary commitment news significantly improved after the workshop when compared to before (3.0 vs. 3.7 for pre- and postworkshop surveys, respectively). Residents trended toward intending to make more changes to their approach after the workshop when compared to before (2.2 vs. 2.6, respectively). Feedback on the didactic and workshop were largely positive. **Discussion:** To our knowledge, our intervention is the first designed specifically to teach psychiatry residents how to communicate to patients that they are being involuntarily committed to emergent psychiatric treatment. This educational model has potential for improving resident skills and confidence in having difficult conversations around involuntary commitment.

Keywords
Difficult News, Involuntary Commitment, Patient Communication, Psychiatry Residency, Psychiatry, Simulation, Standardized Patient

Educational Objectives

By the end of this activity, learners will be able to:

1. Develop greater comfort with their ability to discuss need for involuntary commitment with their patients.
2. Consider their current approach to involuntary commitment conversations and intent to change.
3. Demonstrate delivering news to a patient that they are being involuntary committed for psychiatric treatment.

Introduction

Delivering difficult news can be distressing to physicians and patients and may result in either strengthening or weakening the patient-physician relationship.[1-3] Studies have shown that physicians' skills in delivering difficult news are often suboptimal,[4,5] though they can be effectively taught.[6-9]

Often, psychiatrists must communicate difficult news to particularly vulnerable patients. Knowledge and skill gaps exist among psychiatrists' ability to do so, which can negatively impact the health care experience of patients and their families. These gaps may compromise transparency and, consequently, patients' trust and the therapeutic relationship.[10,11] Despite the importance of communication, standardized training modules for developing communication skills, particularly around difficult conversations, are lacking. One group uses role-playing to provide training on how to share a diagnosis of schizophrenia.[12,13] Additionally, the SPIKES protocol, a protocol on sharing bad news originally designed for oncologists to share cancer diagnoses, is a commonly used teaching guide for difficult conversations.[7,14,15]

One important and often difficult conversation common in psychiatry is communication regarding the need for involuntarily

Citation:
Hsiung K, Skaug L, Daunis D. Communicating a plan for involuntary psychiatric admission: a standardized patient workshop intervention for general psychiatry residents. *MedEdPORTAL*. 2023;19:11355.
https://doi.org/10.15766/mep_2374-8265.11355

Copyright © 2023 Hsiung et al. This is an open-access publication distributed under the terms of the Creative Commons Attribution license.

commitment to psychiatric treatment. Involuntary commitment is the process by which a patient is admitted to a psychiatric facility due to concerns for immediate substantial likelihood of serious harm as a result of a mental illness or emotional disturbance, either against a patient's expressed wish or when the patient is deemed unable to make the decision for admission him- or herself. Maintenance of a therapeutic alliance is paramount to psychiatric treatment, and informing a patient that some individual rights are being restricted can feel challenging and distressing. It can be particularly difficult if the patient is suffering from severe mental illness impacting insight, judgment, and emotion regulation. Furthermore, such a decision is fraught with ethical, personal, cultural, and moral conflicts,[16] and thus, psychiatrists may feel conflicted in both making and communicating the decision. To our knowledge, there is little, if any, literature on communication skills training for sharing news with a patient that they are being involuntarily committed into a psychiatric facility, including in *MedEdPORTAL*. While there is formal education and training regarding when involuntary commitment should be considered or the ethical considerations in involuntary commitment, it is our experience that training on how to effectively communicate the need for involuntary commitment is inconsistent, mostly occurring through unstructured observed interactions in acute medical or psychiatric settings, as is the case with our institution.

Table. Population Demographics

Demographic	Needs Assessment Survey (n = 23)	Presurvey (n = 15)	Postsurvey (n = 12)
Year			
PGY 1	7 (30%)	5 (33%)	5 (42%)
PGY 2	6 (26%)	4 (27%)	3 (25%)
PGY 3	5 (22%)	2 (13%)	1 (8%)
PGY 4	5 (22%)	4 (27%)	3 (25%)
Gender			
Female	10 (44%)	4 (27%)	3 (25%)
Male	13 (56%)	11 (73%)	9 (75%)
What future practice setting do you envision yourself in? (Choose all that apply.)			
Outpatient	9		
Inpatient	8		
Emergency	3		
Consult	2		
Community	5		
>50% nonclinical (e.g., administration, research, education)	2		
Undecided	9		
Other	1		
Have you ever had formal teaching on how to tell a patient that they are involuntarily committed?			
Yes	2 (9%)		
No	21 (91%)		
Have you ever purposely avoided telling a patient they are involuntarily committed because you were afraid of how they would react?			
Yes	9 (39%)		
No	14 (61%)		

To assess the scope of this learning gap in psychiatric training, we designed and distributed a needs assessment survey to general psychiatry residents at Vanderbilt University Medical Center in July and August 2021 (Appendix A). Out of 23 responses, all residents except two (91%) had never received formal teaching on how to tell patients they were being involuntarily committed, clearly indicating the lack of existing instruction on such conversations. Three-fourths of residents (n = 17) rated their comfort in their ability to tell a patient that they were being involuntarily committed as 3 or less out of 5 (1 = *not at all comfortable,* 5 = *very comfortable*). Nine residents (39%) indicated that they had avoided telling a patient that they were being involuntarily committed due to fear of how they would react (Table). Residents of all years feared that discussions about involuntary commitment would lead to patient violence or agitation (60%-69%) and disliked giving disappointing news (44%-58%; Figure 1). Three open-ended responses were received regarding what residents felt uncomfortable about: "What to do when patients become agitated"; "In wrong context, strapped for time, I deliver the news, potentiate agitation, then have to leave and my colleagues have to deal with the consequences"; and "My main concern is that many patients are not in the current state to be accepting of this information and the issues with agitation or violence can be difficult to manage for all team members." Overall, the survey indicated a discomfort across all years of training with sharing involuntary commitment news with patients related to fear of patient agitation.

While patient agitation cannot be prevented, increased preparation can help improve confidence and reduce distress around these conversations. Thus, we designed a workshop for psychiatry residents on how to communicate to a patient the plan for involuntary commitment to a psychiatric hospital. We created a protocol to guide residents on the structure of such a conversation based on clinical experience and review of effective communication practices. Residents first attended a didactic session on the protocol, followed by a 1-hour standardized patient (SP) workshop 1 week later. We used SPs as the primary instructional modality for the workshop as they have had positive outcomes in other communication skills trainings.[6,12,15,17-19] Recognizing that there are various acceptable ways of effective communication, our intention was to provide a suggested structure for the conversation to build resident confidence and decrease discomfort, as well as an opportunity to put it into practice. Emphasis for our activity was placed on teaching

Copyright © 2023 Hsiung et al. This is an open-access publication distributed under the terms of the Creative Commons Attribution license.

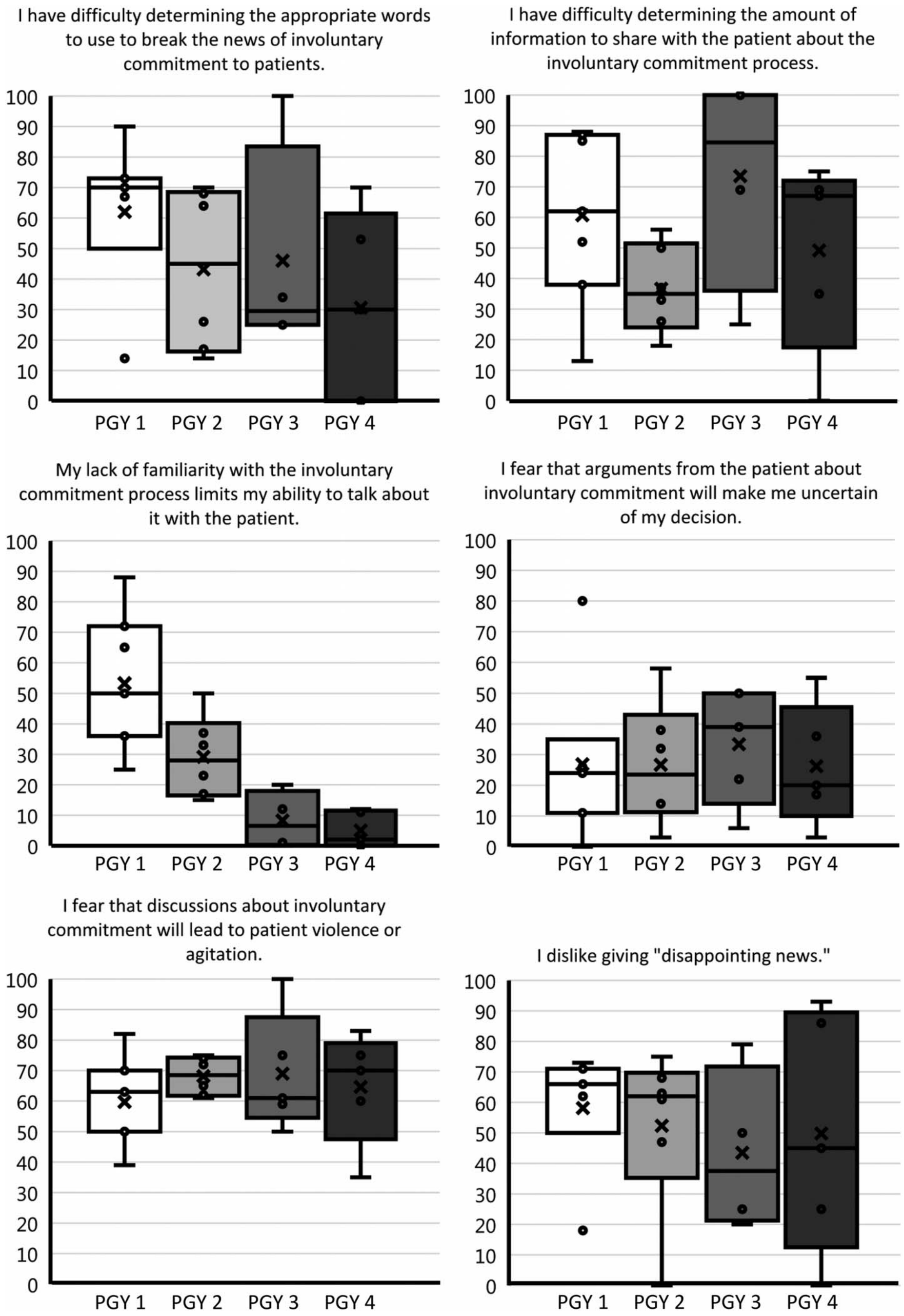

Figure 1. Distribution of needs assessment survey results by year. Participants rated each statement on a scale from 0 to 100, with 100 meaning absolutely true. In the boxes, x = mean, and the open circle (◦) = data point.

over assessment; the latter was intended to be low stakes and formative.

Methods

The involuntary commitment workshop was included as a voluntary addition to the curriculum of the general psychiatry residency program at Vanderbilt University Medical Center in 2021. Target learners included all general psychiatry residents across all 4 years of the program, for a maximum of 33 learners. Prerequisite knowledge included knowing the involuntary commitment criteria of the state of Tennessee, as well as conducting a basic psychiatric interview.

Copyright © 2023 Hsiung et al. This is an open-access publication distributed under the terms of the Creative Commons Attribution license.

Protocol Design

Unstructured interviews were held with two faculty members from the emergency psychiatry and consultation-liaison psychiatry departments. Data were consolidated into a draft protocol, which was then circulated to the faculty who had originally been interviewed, as well as two additional faculty members, for feedback and refining. Edits received after the initial draft protocol were minimal, suggesting a high level of agreement with the protocol steps among all faculty members. The protocol consisted of five steps, plus a preprotocol step 0: (0) initial intake; (1) preparation and safety; (2) summarize concerns and state rationale; (3) be transparent and clearly state involuntary commitment decision; (4) listen, emphasize, and reemphasize the decision; and (5) postconversation communication. The acronym P.S.TLC was used for ease of remembering the protocol steps. For details of the protocol, see Appendix B.

Implementation

One week before the workshop (October 2021), a 1-hour didactic was held as part of the regular weekly resident education schedule. Results of the needs assessment survey were shared, residents were taught the protocol, and two expert discussants shared their experience and answered questions. Residents were emailed a copy of the presentation as well as the scenario prompts to prepare if desired.

Residents were assigned to attend 1 of 2 workshop days, one for PGY 1 and PGY 4 residents and the other for PGY 2 and PGY 3 residents. A link to the preworkshop survey was emailed the day before each workshop, and paper copies were also brought to the workshop. The workshop took place in a large conference room and one smaller classroom-sized overflow room, with chairs arranged in small groups spread out among the space. No additional equipment was required.

At the workshop, residents were divided into groups of two to three per SP. An upper-level resident (i.e., PGY 3 or PGY 4) was assigned to each group where possible. The first 5 minutes were used to fill out the preworkshop survey for those who had not done so online. The remaining time was broken into three 14-minute scenarios, with 2 minutes given to read the scenario, 8 minutes to conduct the encounter with the SP, and 4 minutes for feedback. One minute was allotted for transition between rotations. All residents had the opportunity to perform an encounter. Remaining residents in each encounter observed alongside and used a protocol feedback checklist to deliver feedback along with the SP after the scenario. Two facilitators (Kimberly Hsiung, Daniel Daunis) were present to keep track of time, help manage the rotation of groups during transitions, and guide feedback discussions if needed. After all three scenarios, residents filled out the postworkshop survey. A link to the postworkshop survey was also emailed to all residents who attended. The vast majority of surveys were filled out at the workshop.

The first scenario featured a 50-year-old female with a history of bipolar I disorder brought in by police due to bizarre behavior, with concern for acute mania. The second scenario depicted a 60-year-old female with a past medical history of multiple strokes, brought in by her husband for worsening delusions that the CIA and FBI were conspiring against her. The third scenario featured a 20-year-old male brought in after overdosing on diphenhydramine tablets as a suicide attempt. All scenarios were modified for generalized use; door prompts are available in Appendix C.

SP Recruitment and Training

SPs were recruited and trained through our institution's Center for Experiential Learning and Assessment. Training typically spanned 1-2 days and consisted of reviewing case content and rehearsing case scenarios. SPs observed one another, and feedback was elicited from them as well as from the SP trainer (Laura Skaug). Case content for SPs was modified to fit the *MedEdPORTAL* standardized case development tool and is available in Appendix D.

Assessment

Postdidactic feedback survey: Since the didactic was incorporated into the regular weekly resident educational schedule, a standard online feedback survey that typically followed regular educational didactics was used. The survey consisted of two open-ended questions, one asking for feedback regarding the quality of written handouts, and the second asking for feedback regarding the presentation.

Preworkshop survey: Outside of demographic information, the preworkshop survey consisted of two questions: "How comfortable are you with your ability to deliver news to a patient that they are being involuntarily committed to [the psychiatric hospital]?" and "After learning the protocol steps discussed in last week's didactic, how much do you intend to change your approach to involuntary commitment conversations going forward?" Response options were 5-point Likert scales (Appendix E).

Protocol feedback checklist: To facilitate the peer feedback session, participants were given a feedback checklist with the steps of the P.S.TLC protocol and space for notes as a structured guide. There was no reliability testing to assess the feedback

Copyright © 2023 Hsiung et al. This is an open-access publication distributed under the terms of the Creative Commons Attribution license.

given on the protocol steps. This was because the emphasis of the feedback session was more on having learners of varying levels share experiences and ideas and less on strictly following the steps of the protocol, which had not been rigorously validated (Appendix F).

Postworkshop survey: The postworkshop survey again asked for comfort level and intent to change approach with the same 5-point Likert-scale response options. Including these two questions in both pre- and postworkshop surveys allowed us to measure the extent to which we met Educational Objectives 1 and 2. The survey also provided a space for open-ended and rated feedback on various aspects of the workshop. Finally, participants were asked to share a learning point from the workshop experience (Appendix G).

Statistics

All data from surveys were collected and organized using Vanderbilt REDCap.[20] To assess change in comfort level and intent to change approach pre- and postworkshop, simple summary statistics were used. Data were not paired.

Results

Pre- Versus Postworkshop Survey Comparisons

Residents' perceived comfort level in their ability to deliver involuntary commitment news improved after the workshop when compared to before it (3.0 vs. 3.7 for pre- and postworkshop surveys, respectively). There was a trend toward residents intending to make more changes to their approach after the workshop when compared to before (2.2 vs. 2.6 for pre- and postworkshop surveys, respectively). Figure 2 shows response breakdowns.

Didactic and Workshop Feedback

Feedback on the didactic was overall positive. Many residents commented that they found the didactic helpful and an important topic to review. Some comments expressed appreciation for seeing what other residents struggled with and the progression of these struggles over time.

Feedback on the workshop was overall also positive, with 11 out of 12 residents rating the workshop scenarios as excellent and 10 out of 11 residents believing that the training should be repeated next year in some fashion. Residents shared several learning points, including empathic listening, the importance of preparation, and knowing when to terminate the conversation (Figure 3).

Discussion

Informing patients that they are being involuntarily committed to emergent psychiatric treatment can be uncomfortable. Our needs assessment survey results revealed that among general psychiatry residents, there was a persistent fear of patient agitation precipitated by these conversations. To our knowledge, our intervention is the first protocol and training designed specifically to teach psychiatry residents how to communicate to patients that they are being involuntarily committed to emergent psychiatric treatment.

Our results show that after our workshop intervention, residents felt more comfortable in delivering involuntary commitment news than before. Results also reveal a trend toward residents intending to make more changes to their approach after the intervention than before, showing that the intervention may have inspired a change in practice. Our qualitative feedback indicates that our intervention, particularly its use of SPs, was generally well received by residents, with an overwhelming majority hoping to have this workshop again next year.

Reflecting on the overall execution of our work, we find there are several valuable takeaways. Distributing the needs assessment survey to the residents and sharing its results with them played

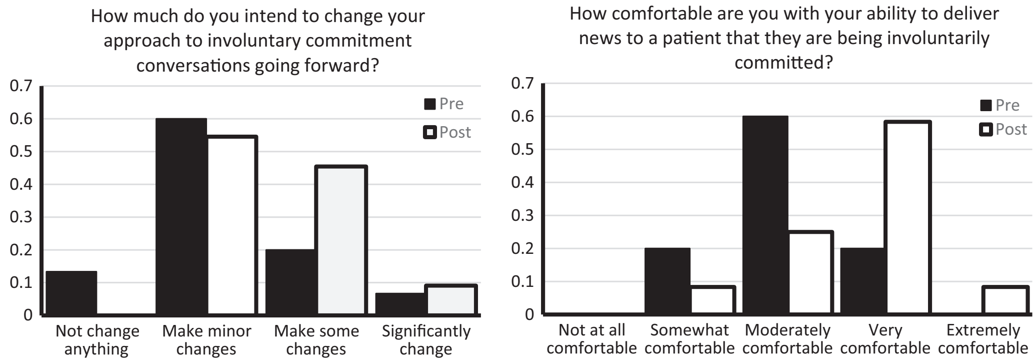

Figure 2. Pre- and postworkshop survey results on intent to change approach and comfort with delivering involuntary commitment news.

Copyright © 2023 Hsiung et al. This is an open-access publication distributed under the terms of the Creative Commons Attribution license.

Didactic Feedback			
"Very well done and a needed addition to our didactics."			
"I think it's a helpful thing to review and definitely will help guide how I navigate these situations in the future."			
"I really appreciated the data presentation from the survey. The tips given were easy to remember and implement."			
"Really enjoyed this presentation as a scaffolding for difficult conversations."			
"Well organized and very well supported by excellent discussants; overall very helpful."			
"The presentation was very helpful. I especially appreciated seeing the data, which helped me to understand which parts of involuntary commitment may become easier and which won't."			
"Excellent presentation and liked seeing what we struggle with and where growth might happen naturally in our training (though we could lessen the time spent floundering) and where we need to stimulate growth otherwise we leave without having developed comfort with a particular process of the talking about involuntary commitment."			
**Workshop Feedback**			
	**Protocol Steps**	**Distribution of Workshop Time**	**Workshop Scenarios**
Excellent	8	8	11
Good	4	4	1
Fair	0	0	0
Poor	0	0	0
**What is your learning point/takeaway from this workshop experience?**			
"I think discussing the cases generally and with different classes of residents was the best part of the seminar."			
"Prep, consistency, and use protocol."			
"Empathetic statements go a long way."			
"Finding a good stopping point: avoiding going in circles. Usually after you've listened/empathized then restated."			
"Empathetic listening and being clear with decision."			
"Knowing when to terminate an unproductive conversation after involuntary commitment."			
"I will try to focus on eliciting the patient's concerns about being in the hospital and addressing those."			
"Have a plan!"			
"Empathy and openness are important."			

Figure 3. Involuntary commitment didactic and workshop feedback.

a vital role in revealing the shared discomfort in communicating involuntary commitment decisions, as well as inspiring buy-in for the need for specific training. Residents expressed a sense of relatedness in seeing the data reflecting the experience of their peers and shared that having a guide for such conversations was an important part of training.

The didactic was crucial in providing residents a tangible framework for the conversation with introducing the P.S.TLC protocol and in preparing them for the hands-on workshop to follow. Our time was restricted to an hour in order to feasibly integrate the workshop into our institution's regular educational schedule; additional time would have allowed an opportunity for more interactive learning, such as simple role-plays to practice each step of the protocol, and/or space for residents to share experiences, concerns, advice, and support with one another, which could be a powerful part of learning for a conversation that can be uncomfortable and distressing.

Regarding the workshop, residents specifically appreciated the use of SPs in making the scenarios appear as realistic as possible. Having a mixture of residents in different stages of training in the same group was also seen as a valuable learning opportunity, with the more experienced residents serving as an example to the more junior ones. Attendance was small overall, which limited interpretation of the impact of our findings, although it did favor the PGY 1 residents, who appear to have benefited most from this intervention. We also later discovered that PGY 3 residents had difficulty getting to the workshop in person due to needing travel time between clinics, which explained their limited attendance. Based on this feedback, we implemented a second iteration the following year targeted to the PGY 1 class only. A PGY 4 chief resident joined each small group to perform an encounter as an example for the PGY 1 residents and also to gain supervisory experience for themselves by facilitating the feedback discussion. Attendance was promoted in this second iteration by making the workshop mandatory.

Copyright © 2023 Hsiung et al. This is an open-access publication distributed under the terms of the Creative Commons Attribution license.

The timing of the workshop in the academic year was important; being a few months into the year, the PGY 1s had experienced the discomfort of involuntary commitment conversations by this point and could see the clinical relevance of the workshop. Our intervention was minimally resource intensive which would make it feasible to reproduce—it was easily introduced into the regular education schedule, and the SPs and services were all free of charge.

Our work has several limitations. First, we recognize the lack of a formal, rigorous method to design and validate the P.S.TLC protocol. Our methods were largely empirical, with input received from only a select population in a single academic institution. Methods of handling agitation concerns may vary depending on local/regional resources, systems of care, and culture. Nevertheless, themes of conveying transparency, genuine concern, and empathic listening are universal in effective communication and likely would vary little if the protocol is developed in a different setting. Finally, our evaluation approach only measures outcomes immediately following our intervention. An outcome measure that may have greater validity in measuring direct impact to patient care would be to assess resident comfort after encountering real-life patient scenarios.

Learner feedback indicated a high interest in having this intervention again in the future. In light of this feedback, there are several potential directions for improvement. The didactic session can be expanded to include more in-session practice role-plays, as well as time to explore resident concerns and fears. Also, an important aspect of communicating involuntary commitment is that the provider must be convinced of the patient's need for involuntary commitment and clear on the reasons why; additional educational time preceding this intervention could focus on learning when involuntary commitment is an appropriate decision to make. Such a decision includes ethical and legal considerations, which may also be an important addition to a future iteration of this intervention. Additionally, our assessments did not formally include objective measures of residency competency. Knowing how fluid conversation can be, our intention with this intervention was not to standardize communication, especially after such a short workshop intervention, but to start with targeting resident discomfort by providing a communication framework and practicing in the face of agitated patients. Future direction may include having more objective assessment, for example, assessing learners on completion of the protocol steps. Finally, having a repeat survey to reassess resident comfort level several months following the intervention would help to determine the extent of its lasting impact.

In summary, we identified that psychiatry residents in our training institution found involuntary commitment conversations challenging due to fear of patient agitation. While preventing agitation may not be completely avoidable, explicit training in this area improves resident preparation and confidence in these circumstances. Our intervention was effective in increasing resident comfort around involuntary commitment discussions and in inspiring change to residents' current approach. As a proof-of-concept, integrating the intervention into regular residency educational curricula was shown to be highly feasible. Having this intervention reproduced in other residency programs would offer more insight into its validity in improving resident comfort surrounding delivering involuntary commitment news.

Appendices

A. Needs Assessment Survey.docx
B. PSTLC Protocol.docx
C. Workshop Scenario Door Prompts.docx
D. SP Case Development Tool.docx
E. Preworkshop Survey.docx
F. Protocol Feedback Checklist.docx
G. Postworkshop Survey.docx

All appendices are peer reviewed as integral parts of the Original Publication.

Kimberly Hsiung, MD, MS: Third-Year Resident, Department of Psychiatry and Behavioral Sciences, Vanderbilt University Medical Center; ORCID: https://orcid.org/0000-0001-5568-2019

Laura Skaug, MFA: Senior Standardized Patient Educator, Center for Experiential Learning and Assessment, Vanderbilt University Medical Center

Daniel Daunis, MD: Assistant Professor, Department of Psychiatry and Behavioral Sciences, Vanderbilt University Medical Center

Acknowledgments
The authors would like to acknowledge Eric Rueth, MD, Amanda Wilson, MD, and Jonathan Smith, MD, for their contributions in providing clinical expertise to developing the P.S.TLC protocol.

Disclosures
None to report.

Funding/Support
None to report.

Copyright © 2023 Hsiung et al. This is an open-access publication distributed under the terms of the Creative Commons Attribution license.

Ethical Approval

Reported as not applicable.

References

1. Luff D, Martin EB Jr, Mills K, Mazzola NM, Bell SK, Meyer EC. Clinicians' strategies for managing their emotions during difficult healthcare conversations. *Patient Educ Couns*. 2016;99(9): 1461-1466. https://doi.org/10.1016/j.pec.2016.06.017
2. Atkinson JM. To tell or not to tell the diagnosis of schizophrenia. *J Med Ethics*. 1989;15(1):21-24. https://doi.org/10.1136/jme.15.1.21
3. Roberts CS, Cox CE, Reintgen DS, Baile WF, Gibertini M. Influence of physician communication on newly diagnosed breast patients' psychologic adjustment and decision-making. *Cancer*. 1994;74(S1):336-341. https://doi.org/10.1002/cncr.2820741319
4. Hagerty RG, Butow PN, Ellis PM, et al. Communicating with realism and hope: incurable cancer patients' views on the disclosure of prognosis. *J Clin Oncol*. 2005;23(6):1278-1288. https://doi.org/10.1200/JCO.2005.11.138
5. Tulsky JA. Interventions to enhance communication among patients, providers, and families. *J Palliat Med*. 2005;8(suppl 1):S95-S101. https://doi.org/10.1089/jpm.2005.8.s-95
6. Back AL, Arnold RM, Baile WF, et al. Efficacy of communication skills training for giving bad news and discussing transitions to palliative care. *Arch Intern Med*. 2007;167(5):453-460. https://doi.org/10.1001/archinte.167.5.453
7. Baile WF, Buckman R, Lenzi R, Glober G, Beale EA, Kudelka AP. SPIKES—a six-step protocol for delivering bad news: application to the patient with cancer. *Oncologist*. 2000;5(4):302-311. https://doi.org/10.1634/theoncologist.5-4-302
8. Razavi D, Delvaux N, Marchal S, Bredart A, Farvacques C, Paesmans M. The effects of a 24-h psychological training program on attitudes, communication skills and occupational stress in oncology: a randomised study. *Eur J Cancer*. 1993;29(13):1858-1863. https://doi.org/10.1016/0959-8049(93)90538-Q
9. Razavi D, Merckaert I, Marchal S, et al. How to optimize physicians' communication skills in cancer care: results of a randomized study assessing the usefulness of posttraining consolidation workshops. *J Clin Oncol*. 2003;21(16):3141-3149. https://doi.org/10.1200/JCO.2003.08.031
10. Mitchell AJ. Reluctance to disclose difficult diagnoses: a narrative review comparing communication by psychiatrists and oncologists. *Support Care Cancer*. 2007;15(7):819-828. https://doi.org/10.1007/s00520-007-0226-y
11. Pestana-Santos A, Loureiro L, Santos V, Carvalho I. Patients with schizophrenia assessing psychiatrists' communication skills. *Psychiatry Res*. 2018;269:13-20. https://doi.org/10.1016/j.psychres.2018.08.040
12. Loughland C, Kelly B, Ditton-Phare P, et al. ComPsych Investigators. Improving clinician competency in communication about schizophrenia: a pilot educational program for psychiatry trainees. *Acad Psychiatry*. 2015;39(2):160-164. https://doi.org/10.1007/s40596-014-0195-7
13. Ditton-Phare P, Sandhu H, Kelly B, Kissane D, Loughland C. Pilot evaluation of a communication skills training program for psychiatry residents using standardized patient assessment. *Acad Psychiatry*. 2016;40(5):768-775. https://doi.org/10.1007/s40596-016-0560-9
14. Deleva VN, Zigman D. A simulation-based communication skills training in treating first-episode psychosis for psychiatry residents. *Acad Psychiatry*. 2020;44(5):638-639. https://doi.org/10.1007/s40596-020-01287-w
15. Amsalem D, Martin A, Mosheva M, et al. Delivering difficult news: simulation-enhanced training improves psychiatry residents' clinical communication skills. *Front Psychiatry*. 2021;12:649090. https://doi.org/10.3389/fpsyt.2021.649090
16. Nussbaum AM. Held against our wills: reimagining involuntary commitment. *Health Aff (Millwood)*. 2020;39(5):898-901. https://doi.org/10.1377/hlthaff.2019.00765
17. Kiluk JV, Dessureault S, Quinn G. Teaching medical students how to break bad news with standardized patients. *J Cancer Educ*. 2012;27(2):277-280. https://doi.org/10.1007/s13187-012-0312-9
18. Cvengros JA, Behel JM, Finley E, Kravitz R, Grichanik M, Dedhia R. Breaking bad news: a small-group learning module and simulated patient case for preclerkship students. *MedEdPORTAL*. 2016;12:10505. https://doi.org/10.15766/mep_2374-8265.10505
19. Neale J. What is the evidence for the use of simulation training to teach communication skills in psychiatry? *Evid Based Ment Health*. 2019;22(1):23-25. https://doi.org/10.1136/ebmental-2018-300075
20. Vanderbilt University. REDCap. Accessed August 31, 2023. https://redcap.vanderbilt.edu

Received: August 28, 2022
Accepted: July 26, 2023
Published: October 17, 2023

Copyright © 2023 Hsiung et al. This is an open-access publication distributed under the terms of the Creative Commons Attribution license.
